# Biographical Feature: Dr. Frederick A. Murphy—a tribute to a pioneer in virology

**DOI:** 10.1128/jvi.01078-26

**Published:** 2026-08-11

**Authors:** Michael D. Lairmore

**Affiliations:** 1 Department of Pathology, Microbiology, and Immunology, Weill School of Veterinary Medicine, University of California, Davis 240470, Davis, California, USA; Dartmouth College Geisel School of Medicine, Hanover, New Hampshire, USA

## TEXT

**Figure:**
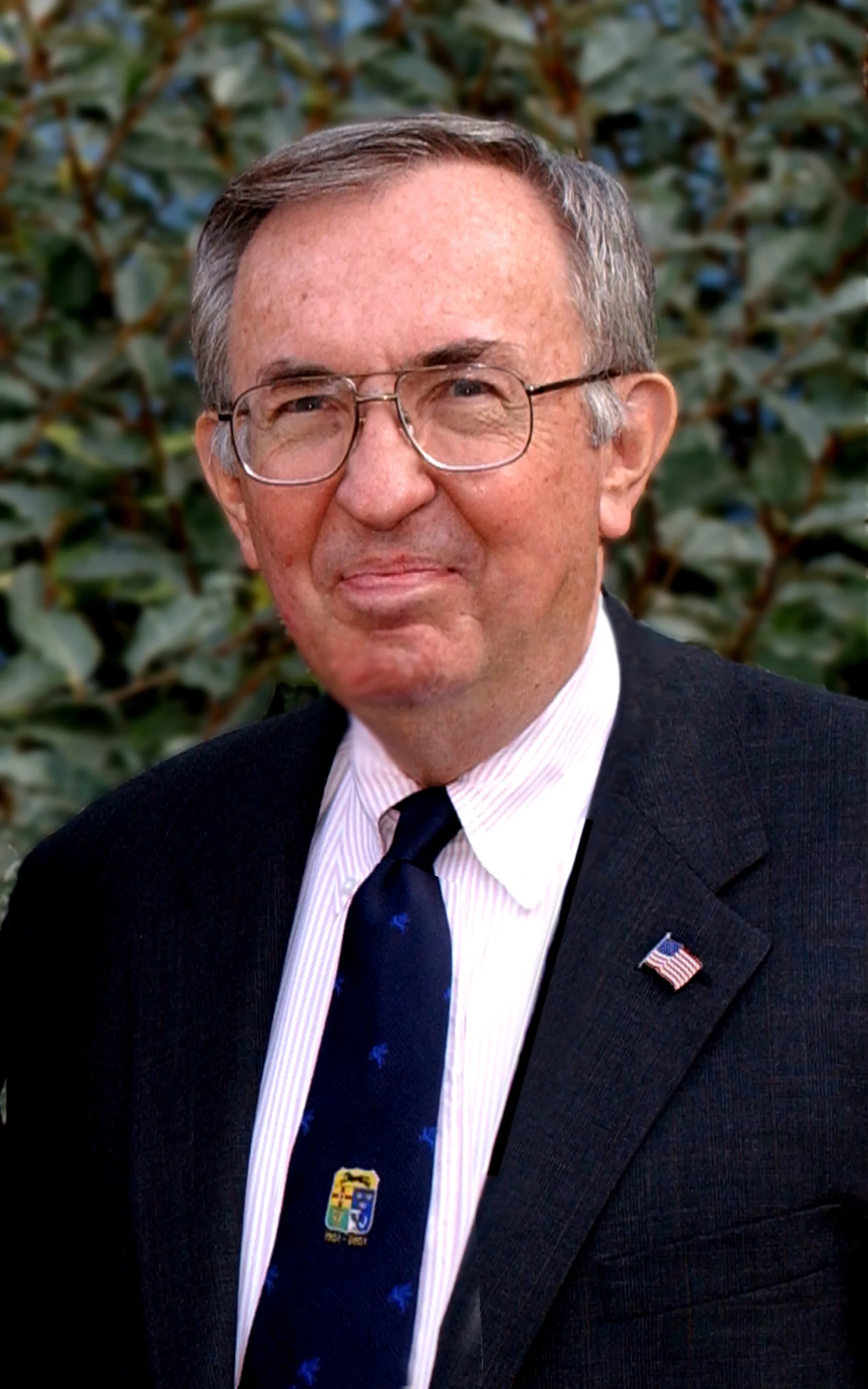


Dr. Frederick A. Murphy, Jr., passed away at the age of 92 on June 23, 2026, in Bethesda, Maryland. His legacy includes novel scientific discoveries of virus-cell interaction, iconic images of viral pathogens, and research insights that helped define the pathogenicity of multiple viruses of public health importance. Dr. Murphy was a dynamic leader who positively influenced the careers of colleagues and scientists around the world. As a natural educator, he promoted the career paths of countless mentees who were inspired by his dedication to evidence-based science, curiosity, and spirit of discovery. His pioneering influence helped develop academic and government research enterprises and strengthened the appreciation of how scientific discoveries translate to the benefit of public health and society.

Dr. Murphy was trained as both a veterinarian and an infectious disease scientist. This unique background fit his passion to understand microbial pathogens and became the framework for him to become renowned for his foundational discoveries in virology and the pathogenesis of viral diseases. He earned his DVM from Cornell University and a PhD from the University of California, Davis (UC Davis). His life’s work was defined by a commitment to the “One Medicine” concept (now termed One Health), emphasizing the intimate links between human and animal health. As a natural servant leader, he served as Director of the Centers for Disease Control (CDC)’s National Center for Infectious Diseases, Dean of the School of Veterinary Medicine at UC Davis (now named the Weill School of Veterinary Medicine), and later Professor at the University of Texas Medical Branch in Galveston. Over his prolific career, he authored more than 500 scientific papers, chaired international virology conferences, edited the journal Archives of Virology, and was inducted into the National Academy of Medicine. Throughout his career, Murphy earned numerous honors, including the U.S. Presidential Rank Award and the Penn Vet World Leadership Award.

Murphy is well known for his role in the discovery of the Ebola virus in 1976 while serving as the Chief of the Viral Pathology Branch at the CDC, but that finding was only one of his unique insights into the world of virology. To understand a newly described deadly disease, he used an electron microscope to capture the first iconic images of the Ebola virus, subsequently named as the new family Filoviridae (from the Latin filo, meaning “threadlike”). His research findings, however, advanced our scientific understanding of many other viruses, including rabies, Lassa fever, and Marburg.

Dr. Murphy was enthusiastic about raising national and international awareness of emerging and re-emerging infectious diseases, as well as mentoring younger scientists. This is how we first met, when he recruited me to the CDC upon finishing my PhD at Colorado State University. His engaging personality, knowledge of scientific discovery, and his humanity, like many of his protégés, constructively influenced the course of my career. Many years later, I followed him as Dean of the School of Veterinary Medicine at UC Davis. More importantly, his drive, energy, and scientific knowledge were a model for his trainees, treating us like a family of curious scientists. He consistently led by example and was dedicated to introducing his colleagues and mentees to other famous scientists and the history of major discoveries in virology.

As a dedicated public health leader, Dr. Murphy served as co-chair of the National Research Council Committee and co-led the committee focused on Occupational Health and Safety in the Care and Use of Nonhuman Primates. He also served on the U.S. Department of Health and Human Services Secretary’s Council on Public Health Preparedness. When he served as President of the International Committee on Taxonomy of Viruses (ICTV), he oversaw a dramatic expansion in the scope of virus classification. Dr. Murphy was known and recognized for his contributions to science around the world and held memberships in the German Academy of Sciences at Berlin and the USSR Academy of Medical Sciences. He was awarded the K.F. Meyer Gold Headed Cane from the American Veterinary Epidemiology Society (now the American Veterinary One Health Society). Murphy was recognized with honorary doctorates from the University of Turku, Finland, and the University of Guelph, Ontario, Canada.

Dr. Murphy was a highly valued quality scientific writer and became a resolute historian of virology, publishing the visually rich book *The Foundations of Virology*. In the foreword of the book, Fred humbly notes, “This book is an attempt to remember some of the discovers and discoveries, inventor and inventions, and developers and technologies that were seminal in the advance of medical and veterinary virology.” The book illustrates his ethos and respect for basic and applied virology research that elucidated discoveries and concepts to benefit society across the globe.

Beyond his images that helped in the discovery of the Ebola virus, he studied other related viruses such as Marburg and conducted some of the first experimental infections to understand the pathogenesis of the deadly virus. Through his research findings, Dr. Murphy became an international authority on the pathogenesis of rabies and related viruses, including the Lagos bat virus. In collaboration with the local public health officials, he helped assemble and lead a team at the CDC and the U.S. Army that characterized a new Hantavirus strain, later called Sin Nombre virus, following the outbreak that plagued the Four Corners region of the Southwestern United States in 1993. His research extended to various viral causes of encephalitis, including Western equine encephalitis, St. Louis encephalitis, and Venezuelan equine encephalitis. Using electron microscopy as a principal tool, he helped define the pathogenesis of other viruses including Lassa virus, coronaviruses, and vesicular stomatitis virus.

Like many of his mentees, I benefited from Fred’s inspiring leadership and teaching style. He challenged those he trained or supervised to collaborate and use scientific curiosity to forge new ways of understanding whatever pathogen was being investigated. He treated his employees and staff as colleagues who were partners in a quest for new knowledge. His energetic style challenged those he worked with to seek answers critical to understanding how viruses replicated and caused disease. Then, as a true scholar and scientist, driven by evidence-based science, he constantly sought to communicate his findings through high-quality and accessible peer-reviewed publications.

Dr. Murphy was surrounded by his loving family. Fred married his wife Irene in 1960 and shared 40 years of a loving union before her passing in 2000. He is survived by his four sons and their families. He frequently mentioned Irene and his family in endearing terms, citing them as a guidepost for his love, inspiration, beliefs, and hope for the future.

For those of us who had the privilege to know and work with Fred, we also knew him as a humble person, modest in describing his contributions to virology. Dr. Murphy has left a pioneering legacy of discovery, scientific excellence, compassionate interdisciplinary teamwork, and a spirit of generous sharing of knowledge to make the world a better place for his family, colleagues, friends, and society.

